# Geospatial analysis of cesarean section in Iran (2016–2020): exploring clustered patterns and measuring spatial interactions of available health services

**DOI:** 10.1186/s12884-022-04856-z

**Published:** 2022-07-21

**Authors:** Alireza Mohammadi, Elahe Pishgar, Zahra Salari, Behzad Kiani

**Affiliations:** 1 Department of Geography and Urban Planning, Faculty of Social Sciences, University of Mohaghegh Ardabili, Ardabil, Iran; 2 Department of Human Geography, Faculty of Earth Sciences, Shahid Beheshti University, Tehran, Iran; 3 Jahrom University of Medical Sciences, Jahrom, Iran; 4 Department of Medical Informatics, School of Medicine, Mashhad University of Medical Sciences, Mashhad, Iran; 5 Centre de Recherche en Santé Publique, Université de Montréal, 7101, Avenue du Parc, Montréal, Canada

**Keywords:** Cesarean section, Spatiotemporal analysis, Spatial interaction, Spatial epidemiology, Geographical information systems

## Abstract

**Background:**

The lives of babies and mothers are at risk due to the uneven distribution of healthcare facilities required for emergency cesarean sections (CS). However, CS without medical indications might cause complications for mothers and babies, which is a global health problem. Identifying spatiotemporal variations of CS rates in each geographical area could provide helpful information to understand the status of using CS services.

**Methods:**

This cross-sectional study explored spatiotemporal patterns of CS in northeast Iran from 2016 to 2020. Space–time scan statistics and spatial interaction analysis were conducted using geographical information systems to visualize and explore patterns of CS services.

**Results:**

The temporal analysis identified 2017 and 2018 as the statistically significant high clustered times in terms of CS rate. Five purely spatial clusters were identified that were distributed heterogeneously in the study region and included 14 counties. The spatiotemporal analysis identified four clusters that included 13 counties as high-rate areas in different periods. According to spatial interaction analysis, there was a solid spatial concentration of hospital facilities in the political center of the study area. Moreover, a high degree of inequity was observed in spatial accessibility to CS hospitals in the study area.

**Conclusions:**

CS Spatiotemporal clusters in the study area reveal that CS use in different counties among women of childbearing age is significantly different in terms of location and time. This difference might be studied in future research to identify any overutilization of CS or lack of appropriate CS in clustered counties, as both put women at risk. Hospital capacity and distance from population centers to hospitals might play an essential role in CS rate variations and spatial interactions among people and CS facilities. As a result, some healthcare strategies, e.g., building new hospitals and empowering the existing local hospitals to perform CS in areas out of service, might be developed to decline spatial inequity.

**Supplementary Information:**

The online version contains supplementary material available at 10.1186/s12884-022-04856-z.

## Background

Maternal mortality remains a global challenge [1]. Approximately 810 maternal deaths occur due to preventable causes related to pregnancy and childbirth, of which 94% are observed in developing countries [2]. Cesarean section (CS) is often a life-saving intervention for mothers and babies [3]. Inequality in spatial access to CS services significantly increases maternal mortality [4]. However, appropriate spatial access to timely, adequate, and quality CS services can reduce 60% of mortality and complications in mothers and babies [5, 6] in low and low-middle-income countries. On the other hand, CS overutilization by some population groups when it does not have any medical indication is another significant public health problem [7]. According to the World Health Organization (WHO), CS should cover 10% to 15% of all deliveries at most [8, 9]. However, the worldwide CS rate increased from 6.7% in 1990 to 21% in 2021, with 14.3% growth [8], and spatial variations in CS distribution are observed in different geographical areas [10, 11].

Geographical information systems (GIS) are a kind of computer system that integrate both spatial and non-spatial data [12, 13]. Spatial data are related to geography, for example, the locations of women using CS or the hospitals performing CS. Non-spatial data include all of the other data, such as the capacity of hospitals or individual characteristics of women undergoing CS. GIS technology could develop knowledge regarding the spatial distribution of CS cases or the spatial interaction between the women using CS and the hospitals performing CS [14, 15].

The determinants of choosing CS for delivery are multifaceted and complex. The recent scientific literature on why CS is the preferred delivery method could be summarized in four groups of studies. The first group focused on the compulsory causes of CS. They include fear of labor, anxiety for fetal injury/death, doctor’s suggestion, mothers’ previous experiences such as infertility, obstructive labor, anxiety for gynecologic examination, emotional aspects [4, 16], mother’s age, body mass index and her health condition, time of birth, any experience of prior complicated delivery, the weight of baby, pathological placenta, severe bleeding during childbirth, wound infection, hematoma, intestinal obstruction and abnormal prenatal situation [4, 16–24].

The second group of studies focused on socioeconomic factors (e.g., household income, residence address as town or village, education level, and mother’s age at the time of marriage) that could be associated with CS on maternal request (elective CS) [3, 25–32]. For instance, Feng et al. [26] showed that the CS rate in urban areas was three times more than in rural areas, and economic condition was one of the most contributing factors in choosing CS rather than natural childbirth.

Some studies as the third group focused on the importance of accessibility to CS-related facilities in choosing the type of delivery [33–35]. For instance, Kumar et al. [35] showed that with a 1-km increase in distance from an individual’s place to the nearest medical center for CS operation, the probability of choosing CS is declined by 4.4 times. Also, Kesterton et al. [34] showed a 32% increase in referrals to a hospital for CS with each 5-km closer to the hospital. Emily et al. [4] revealed that CS rates increased in Uganda, and there was geographical heterogeneity in CS rates and inequality in CS-related health facilities.

The fourth group of studies used geographical and spatial analysis to explain and interpret CS rates [11, 19]. For instance, Vanderlaan et al. [11] in Georgia identified counties with high-rate CS clusters had significantly lower access to midwives. Furthermore, more deliveries were paid by Medicaid in these counties, and a higher proportion of births for women belonging to racial/ethnic minority groups were more likely to be rural. In another study in Ethiopia, Tegegne et al. [36] showed that CS rates were clustered in areas with a high percentage of urbanization and the highest available medical facilities (e.g., the country’s capital). In another study in Norway, Mannseth et al. [37] showed that high-risk deliveries were concentrated in large hospitals with better services and equipment that were not evenly distributed in different country regions. Consequently, this affected the CS rates spatial distribution.

In terms of CS rates, North-east Iran, including our study area, is one of the regions with high rates of CS, about 43% [38]. Furthermore, the spatial distribution of health services in this region is highly unequal, affecting the health of mothers and children during childbirth [39]. No study in Iran has examined potential geographical variations of CS with a spatiotemporal approach. Therefore, this study aims to analyze the temporal, spatial, and spatiotemporal patterns of CS in northeastern Iran. The study also attempts to visualize available medical services for CS considering distance and service area approaches.

## Methods

### Study region

**Fig. 1:**
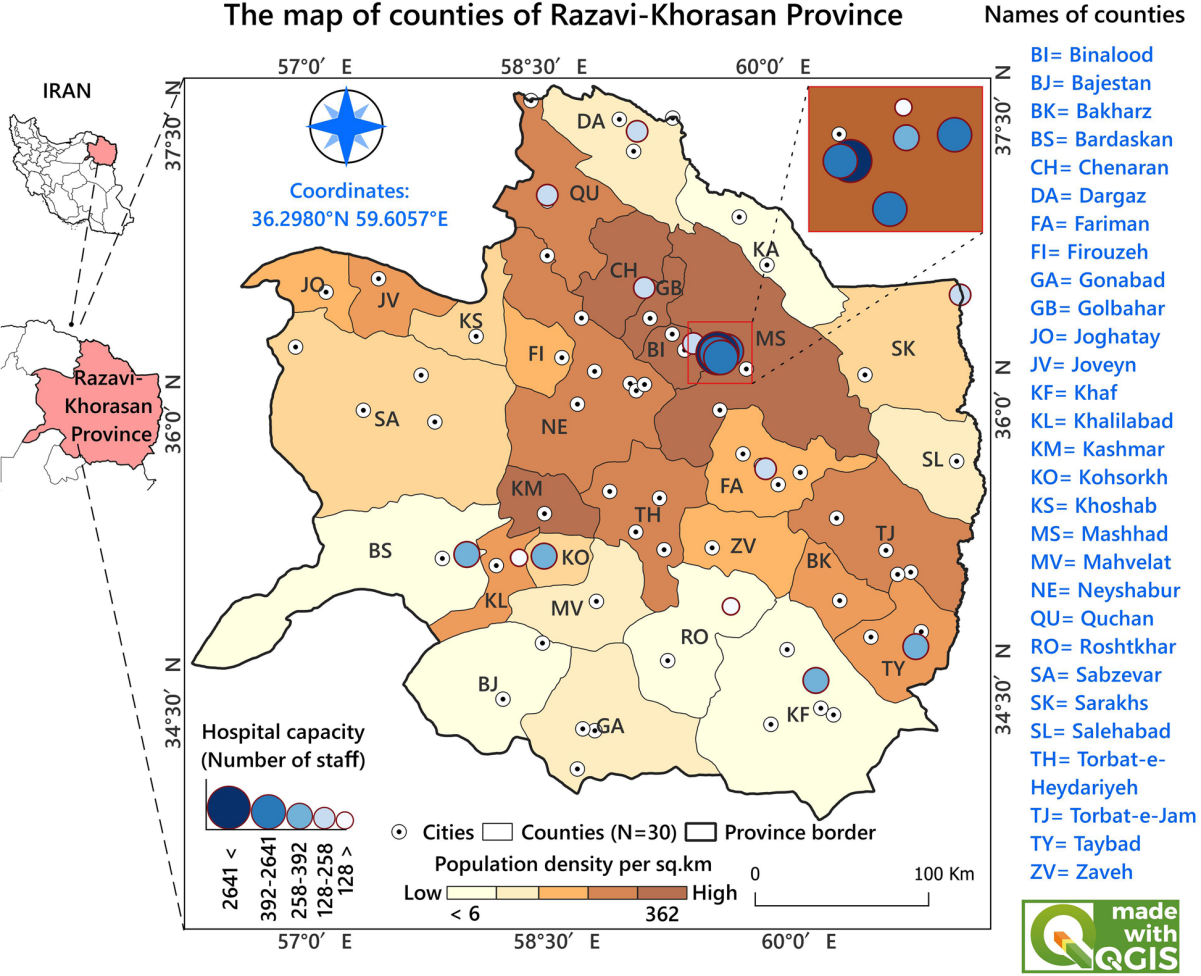
Map of the study area including hospital capacity and population density of different counties

Khorasan-Razavi Province (KRP) is located in North-East Iran at latitude 35.1020° North, longitude 59.1042° East (Fig. 1). KRP covers an area of 119 km^2^, and its population was about 6,435,000 (12.2% of Iran's population) in 2015, of which 75% were located in urban areas. There were 18 public hospitals in the study area (Fig. 1). All hospitals are located in counties’ centers. In Iran, counties are used as the basic unit of socio-economic and health studies, and most statistics are gathered at this level. Furthermore, most health services are concentrated in counties’ main centers. Therefore, pregnant women living in villages or cities refer to these main centers. There were 30 counties in the study region and, we used county level as the scale for spatial analysis. An important fact is that the people in KRP vary with respect to race and ethnicity. This diversity might be because of the vastness of KRP and its geographical and climate variation, a reflection of the situation of Iran as a whole, which makes KRP a representative sample of the whole country.

### Datasets

The total CS data including elective and prescribed CS were obtained from Mashhad University of Medical Sciences, which collects and stores information on pregnancy and births in the study area. The data included 74,291 CS from 1 January 2016 to 31 December 2020 gathered from 18 public hospitals performing CS. Incomplete and invalid data (123 CS cases) were excluded from analyses. To consider participants’ privacy, all personal information of individuals was removed from the records. Information such as age, residence address, hospital name, and admission dates was used. All spatial data layers were projected to WGS 1984 UTM Zone 40 N system. The number of all staff was used as the hospital's capacity. This information was obtained from the Mashhad University of Medical Sciences, which manages all government hospitals in the study area.

### Data analysis

#### Empirical Bayesian Smoothed (EBS) rates

An Empirical Bayes Smoothing (EBS) approach was used to calculate the spatially smoothed rate of CS deliveries. The relevant number of women of childbearing age (between 15–49 years) varies across areas under investigation, so the precision of raw CS rate varies as well [40]. This variance instability requires smoothing, and we used the local EBS technique to reduce the random fluctuations due to the population size of women of childbearing age [41, 42]. To calculate the local EBS CS rate for each county, we considered the number of CS deliveries as the event variable and the total number of all women of childbearing age as the population field.

#### CS rates visualization

Choropleth maps represented the EBS CS rates by four rate classes varying from Low: < 65 to high: ≥ 13,553 per 100,000 women at childbearing age. The rates were classified into four classes based on the equal quantile method. This classification let us compare the rates in different periods and geographical areas.

#### Global spatial autocorrelation

We used Global Moran's Index (GMI) to measure spatial dependency since it is generally more accurate concerning spatial autocorrelation than other metrics [43, 44]. This index measures how one object is similar to others surrounding it. If objects (CS rates in this study at county level) are attracted by each other, it means that the observations are not independent. The "contiguity edges corners" method was used to model spatial relationships, which is suitable for polygon type complications. In this method, polygons that share an edge or a corner will be included in computations for the target polygon [45]. Although GMI statistics is a powerful way to indicate spatial autocorrelation, this method alone cannot identify hidden spatiotemporal patterns and clusters of CS rates over time [46]. To address this limitation, local spatial and spatiotemporal statistics were also used as follows.

#### Spatiotemporal methods

The Scan statistics methodology includes purely temporal analysis, purely spatial analysis, spatiotemporal analysis, and spatiotemporal variation in temporal trends. The methods can detect clusters irrespective of any predefined geographical boundaries in predefined study time [47]. In this study, the retrospective Poisson probability model as a discrete type of scan statistic was used to analyze the rates of CS deliveries in the study region from 2016 through 2020. Time aggregation length was set to 1 year, and the maximum window size of analysis was adjusted to 50%. The null hypothesis of no clusters was rejected at the simulated *p* ≤ *0.05* for the primary (most likely) clusters, and 999 Monte Carlo replications were performed for statistical inference. Statistical details of the results of these methods are given in Tables 1, 2 and 3 of Additional file 1.

#### Interaction analysis

Spatial interaction is the degree of linkage between the origin and the destination [48]. In this study, we used two methods to detect and analyze the spatial interactions among counties centroids and then hospitals based on simplified steps of the gravity model in the GIS environment.

The basic gravity model was used according to Eq. 1 to identify potential spatial interactions among counties (Horner, 2009).

1 $$I_{ij}=\frac{P_iP_j}{d_{ij}^2}$$

where ${I}_{ij}$ is the interaction between locations *i* and *j*, *P*_*i*_ is the hospital capacity at location *i*, *P*_*j*_ is the hospital capacity at location *j*, and *d*_*ij*_ is the distance between locations *i* and *j*. One of the most significant aspects of gravity models (or as they are now known in geography – spatial interaction models) is that they formally specify the relationship between distance and the likelihood of interaction.

In addition to the gravity model, we calculated the CS weighted rate flows for each county using Eq. 2:

2 $$\mathrm{CS}\;\mathrm{weighted}\;\mathrm{rate}\;\mathrm{flow}:\;\frac{CS\;rate_{i,j}}{\underset{}{\sum CS\;rate_{i,j}}}\mathrm \times1$$

where; ${CS rate }_{i,j}$, is the rate of CS per 100 hospital staff as its capacity in the county *i* at hospital *j*. The result is a normalized value between 0 and 1.

#### Software

All scan statistics analyses were conducted using SatScan software (developed by Martin Kulldorff and Information Management Services Inc. Cambridge, Massachusetts, 1997). QGIS software version 3.20, a free and open-source GIS software, was used to visualize the choropleth maps and GeoDa 10.8 to calculate EBS rates.

## Results

### Descriptive analysis

**Fig. 2:**
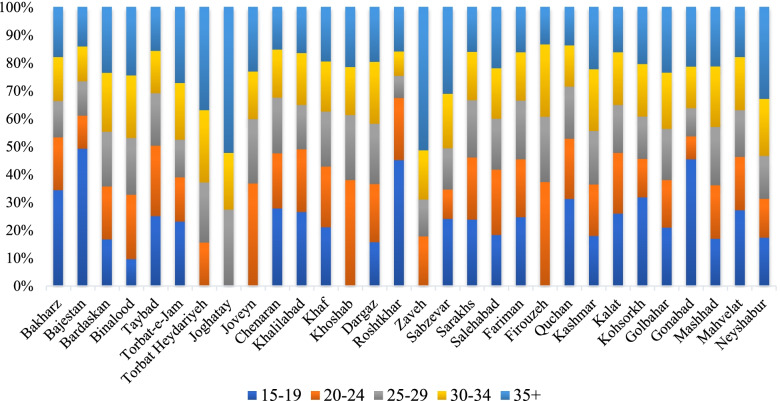
The percentage of CS (%) and mothers' age groups by county

During the study time, from the total 209,935 deliveries performed in public hospitals of KRP, 74,291 deliveries were done by CS (35.39% of the total). The average age of mothers was 32.33 years at the time of CS, but the age followed some geographical variations (Fig. 2). For example, the highest average age was in Joghatai (36 years), and the lowest average was in Firouzeh county (29.20 years). Among the five age groups (15–19, 20–24, 25–29, 30–34, and $\ge$ 35 years) of mothers who had a CS experience, the highest portion of CS (37.64%) was observed in the age group of 35 years and older. The lowest CS rate (1.48%) was related to the age group of 15 to 19 years.

**Fig. 3:**
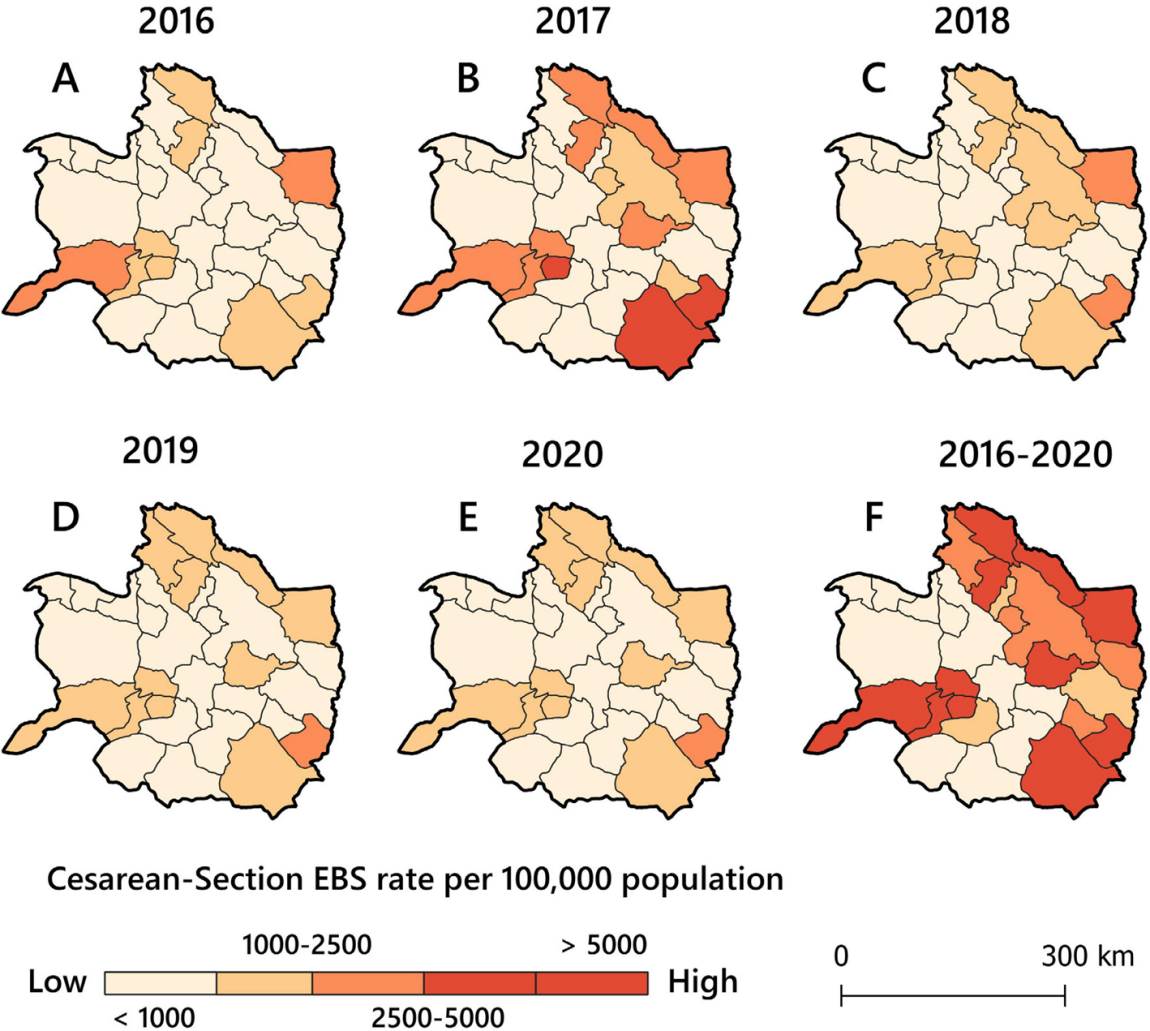
Rate maps of CS deliveries at the county level in KRP, Iran. Dark-red regions represent the highest CS rates, and light-yellow regions represent the lowest CS rates

According to Fig. 3, out of 30 counties, 11 (36.7%) counties experienced high rates (mean = 4,670 and SD = 4,553 per 100,000 women of childbearing age). The overall CS rate decreased from 862 per 100,000 women in 2016 to 844 per 100,000 women in 2020. However, the mean rate of CS was higher in 2017 (1,079 per 100,000 women of childbearing age) among the other years. These results confirmed that most counties with high rates (> 5,000 per 100,000 women of childbearing age) were in the east, southeast, and west. The highest rate was 13,553 per 100,000 women of childbearing age.

### Purely temporal and spatial clusters

**Fig. 4:**
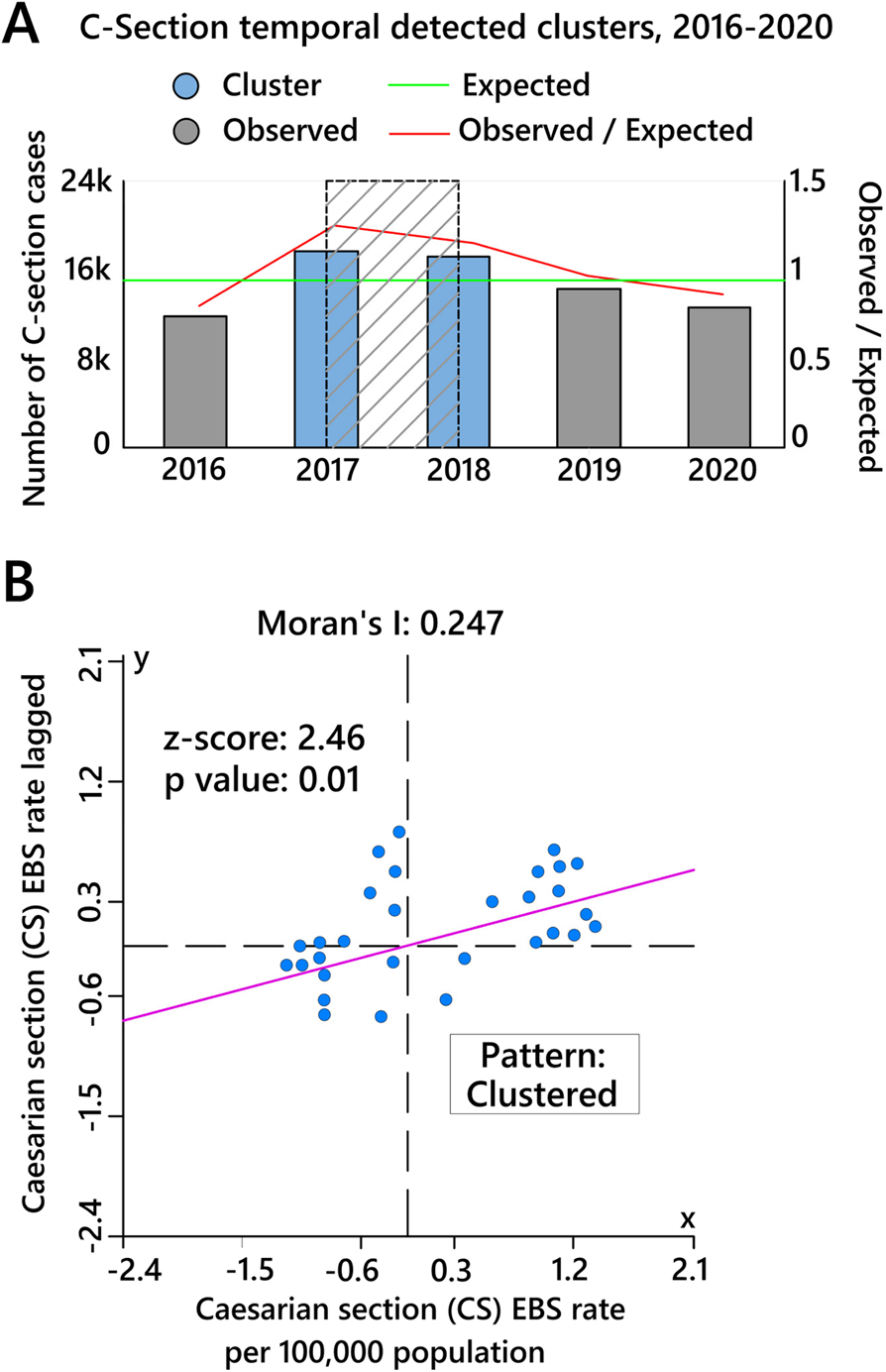
**A** Purely temporal clusters identified by 50% maximum window cluster size in the study region between 2016 and 2020, note: the light blue banded plot area shows significant temporal clusters; **B** Global Moran’s I statistics for EBS rates of CS deliveries per 100,000 women of childbearing age within the study region from 2016 to 2020

**Fig. 5:**
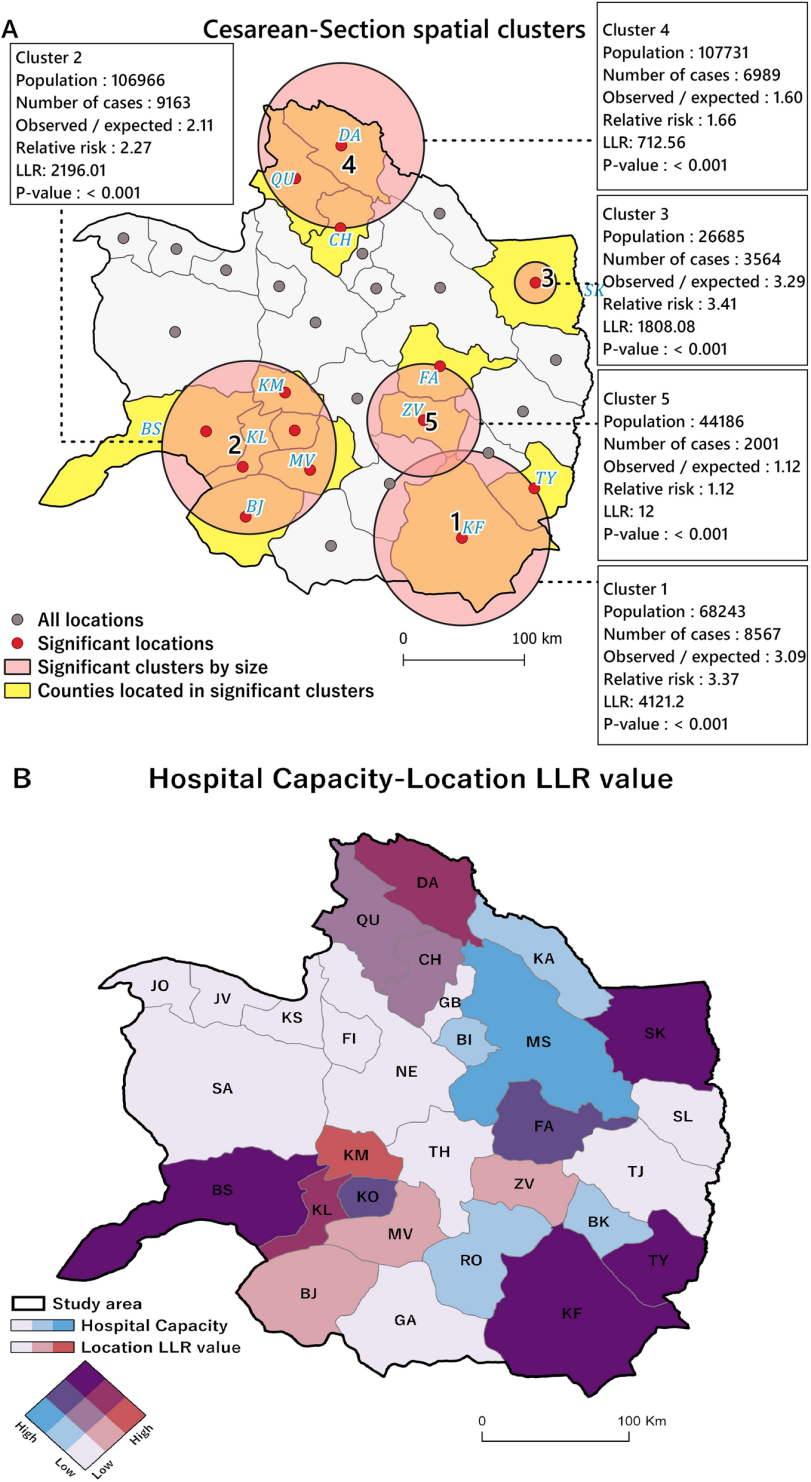
**A** Purely spatial clusters of CS rates identified by the SaTScan approach in the study area; **B** Bivariate choropleth map comparing hospital capacity and LLR score within the study region from 2016 to 2020; shades of purple show significant proportions of both variables

Figure 4A indicates that high-rate time clusters of total CS were predominantly distributed (*OE* = *1.18, RR* = *1.34, LLR* = *799.97, p* < *0.05*) between 2017 and 2018. Global Moran’s *I* statistics for CS deliveries based on EBS rates (*Moran’s I* = *0.247, z-score* = *2.46, p* < *0.05*) revealed that the spatial autocorrelation was significant, and the null hypothesis was rejected as CS incidence rates were spatially clustered (Fig. 4B). Figure 5A shows the purely spatial pattern of CS incidence rates based on the Poisson probability model of scan statistics. Five most likely clusters (*RR* > 1, *p* < 0.05) (hot spots) were identified that were distributed heterogeneously in the study region, and 14 of the 30 counties were located in these five clusters. Figure 5B displays a bivariate choropleth map comparing hospital capacity and LLR scores based on purely spatial clusters of CS rates in the study region. The result shows that the spatial clustering pattern has followed the spatial distribution pattern of hospital services within the study region.

### Spatiotemporal clusters

**Fig. 6:**
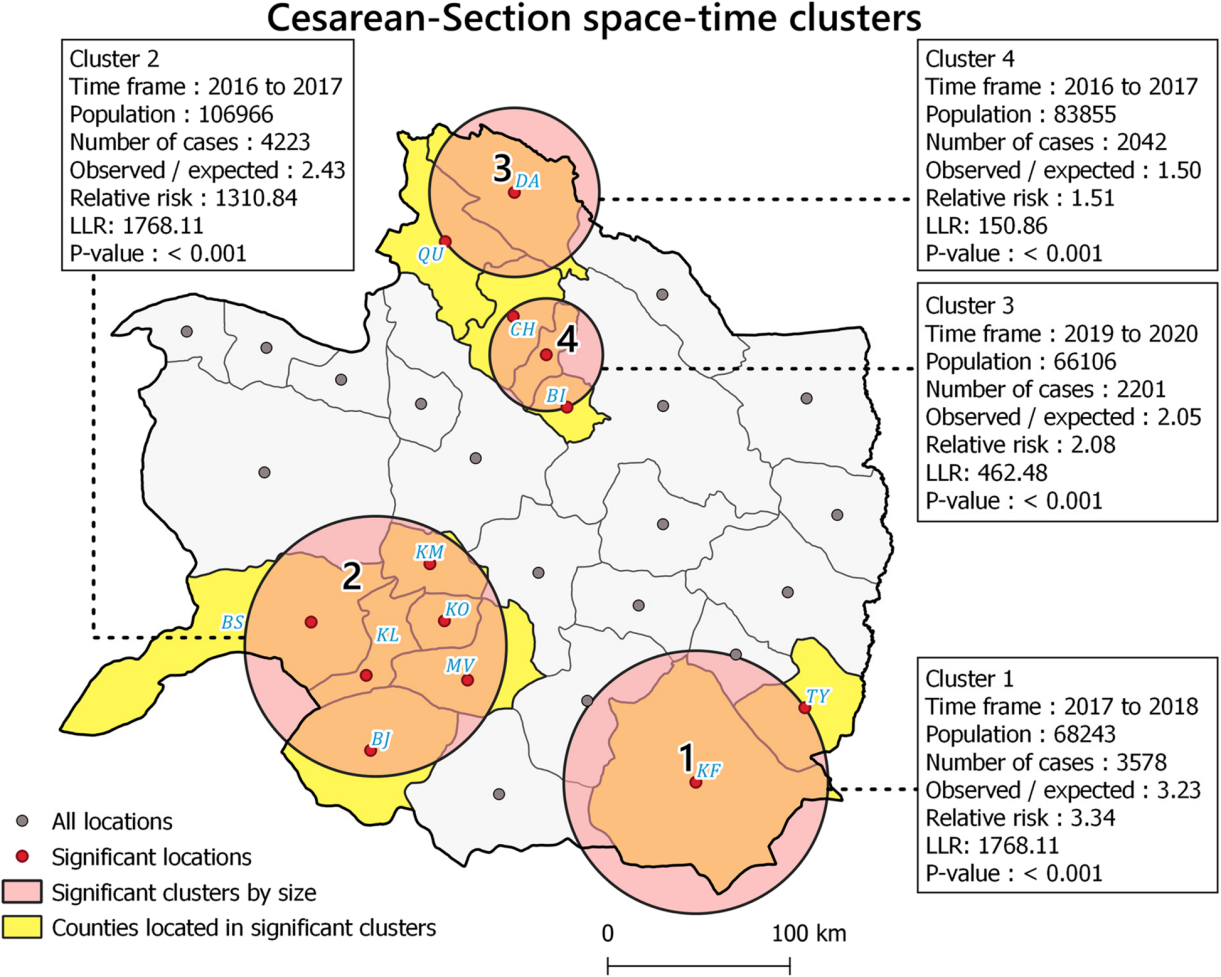
Spatiotemporal cluster identified by SaTScan approach between 2016 and 2020

The study region's statistically significant high-rate CS spatiotemporal clusters were mainly south to north (Fig. 6). Based on the 50% maximum window size, altogether 13 locations (counties) were classified as high-rate areas in different time periods (RR > 1, *p* < 0.05) (Fig. 6). For example, cluster one formed from 2017 to 2018 in the southeast, while cluster three was in the north between 2016 and 2017.

### Spatial variation in temporal trends

**Fig. 7:**
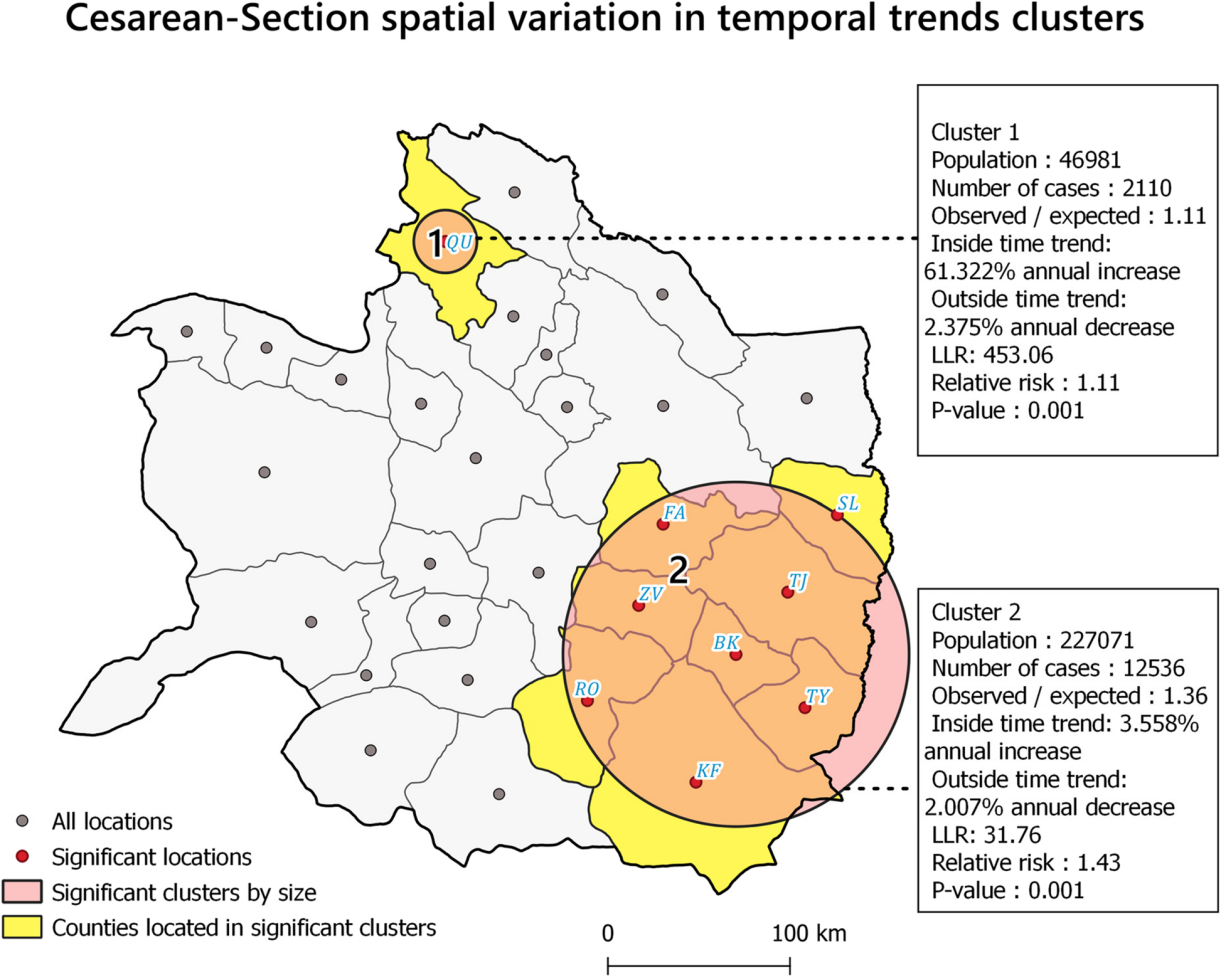
Clusters of spatial variations in temporal trends map

According to Fig. 7, two high-trend CS clusters were found. These clusters included the counties with the highest variations compared to counties inside and outside. The first cluster formed in the north, and the second cluster stood in the southeast of the study area. In the first cluster, one location was in high-trend statistically significant clusters (LRR = 453.01, RR = 1.11, *p-*value < 0.05). In the second cluster, there were eight statistically significant high-trend clusters (LRR = 31.76, RR = 1.43, *p-*value < 0.05). It is clear that CS rates were increasing overall, but not in the same way in all areas. For example, cluster one experienced an average annual growth of 61.3%, but this growth was 3.56% in cluster two.

### Interaction analysis

**Fig. 8:**
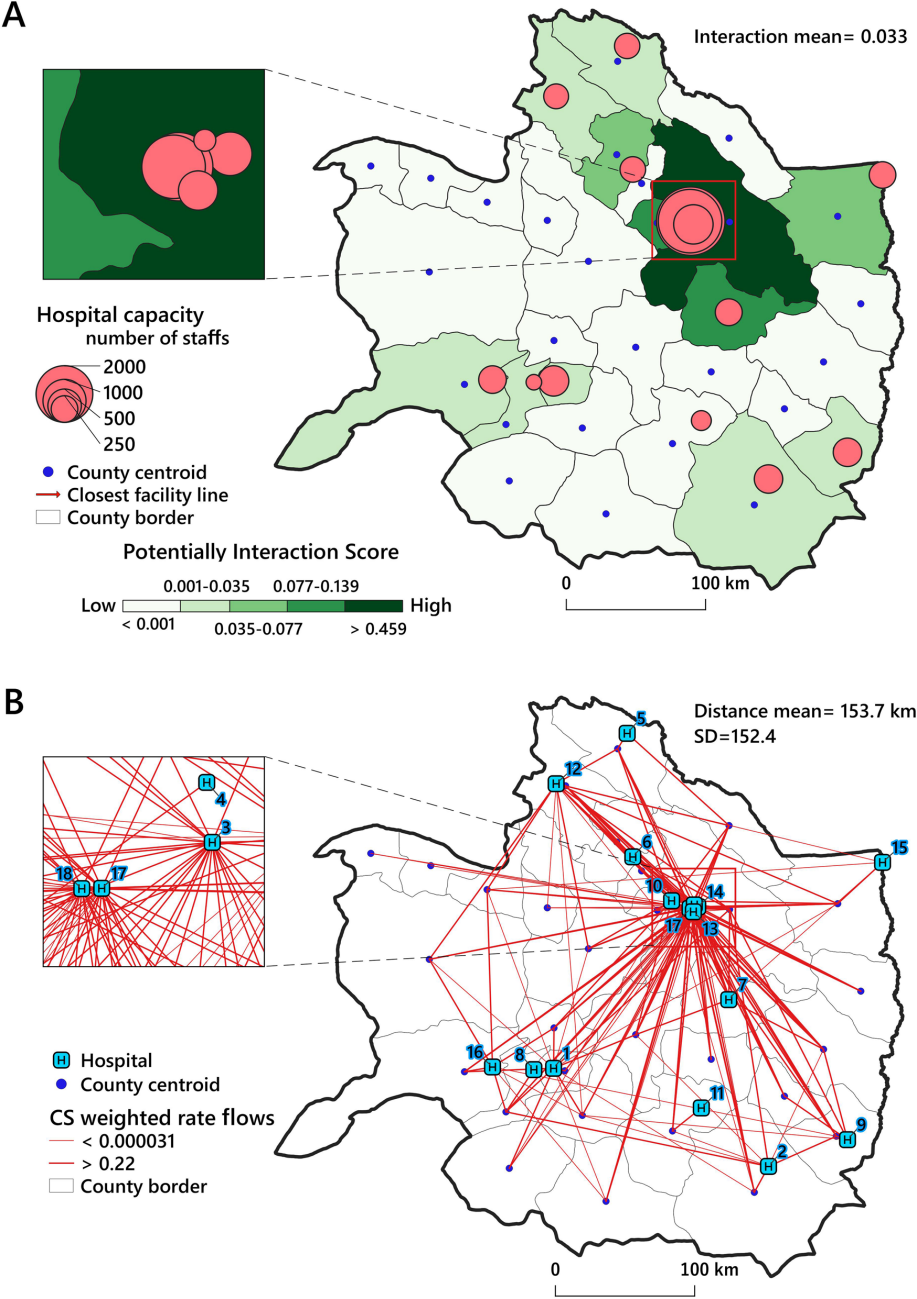
**A** Potential spatial interaction among the counties in the study area. **B** CS weighted rate flows values (red color lines) between origins (counties centroids) and destinations (hospitals)

Figure 8A shows potential spatial interactions score (values normalized between 0 and 1) among 30 counties in the study area based on the “*basic gravity model*” expressed in Eq. 1. According to this map, some regions in the northeast (Mashhad County) have a high potential gravity (potentially integration score > 0.139) to attract CS flows in the study area. Furthermore, it can be found that close distance and health centers' capacity have a direct effect on increasing spatial interactions. The complete results of interaction analysis have been provided in Additional file 2.

The CS weighted rate flows model shows that the intensity of linkages and the capacity of hospitals (Fig. 8B) have a profound effect on the CS flows in the study region.

**Fig. 9:**
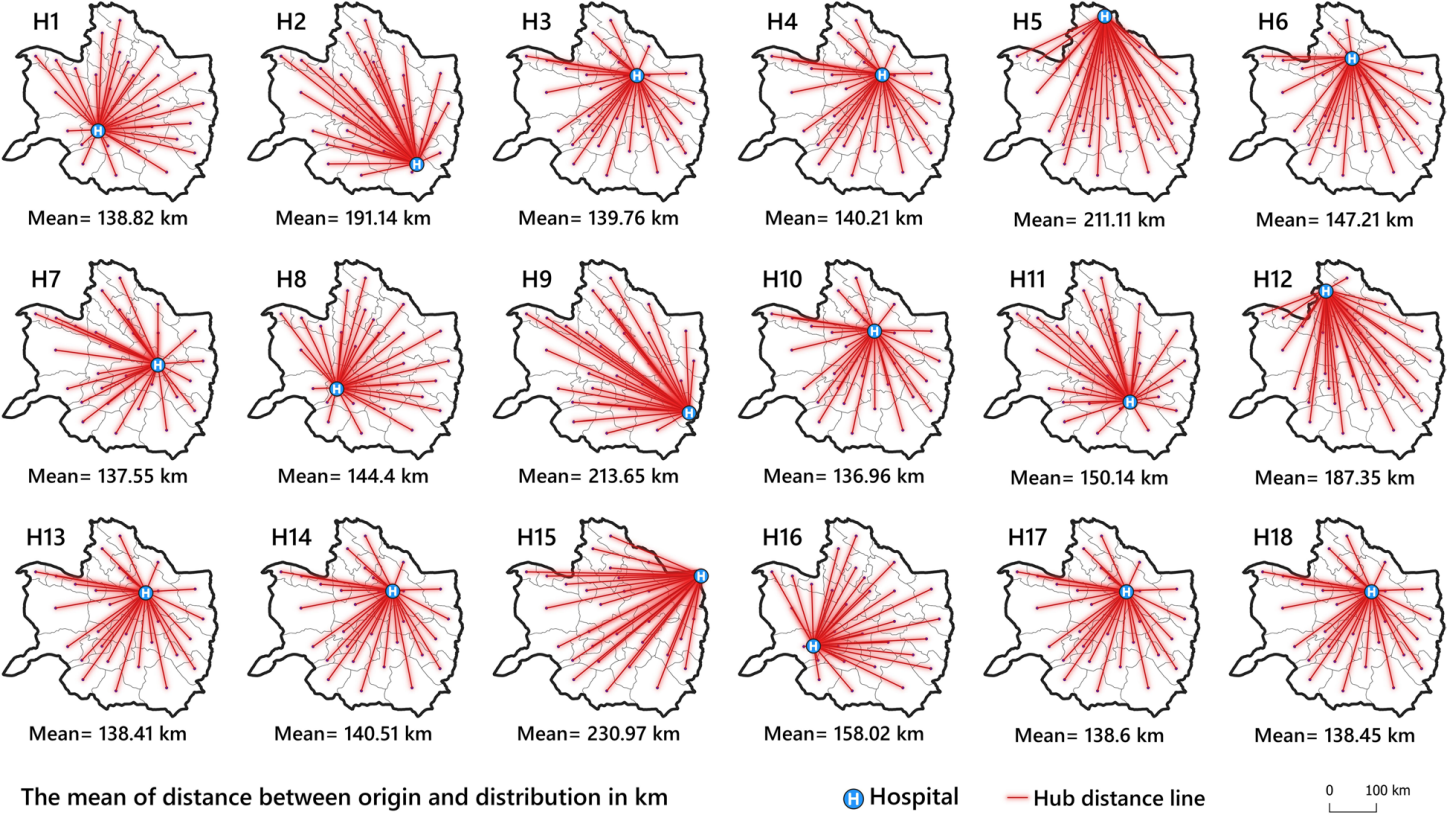
Average distance between each public hospital (destinations) and county centroids (origins) in km in the study area. Note: symbols H1 to H18 indicate the identification number of the hospitals

Figure 9 shows the distance between origins and destinations in km. According to the analysis, the mean distance between county centers and hospitals in the entire study region was 153.7 km. The results show that most hospitals located in the northeast of study area have a shorter mean distance to county centroids (mean distance < 153.7 km). For example, hospitals 3,4,13,14,17, and 18, were at an average distance less than 139.32 km from the county (population centers) centroids and they were spatially most accessible.

## Discussion

The present study provided a spatiotemporal analysis of CS rates in northeastern Iran between 2016 and 2020. Our results based on purely spatial analysis of EBS rates showed significant differences among diverse counties regarding CS rates, which led to spatial heterogeneity in the distribution of CS rates in the study region and study time. Moreover, Global Moran’s I autocorrelation test results confirmed the existence of spatial dependency in the study area. The purely spatial analysis was used to find the precise location of clusters and revealed five clusters in the study area (Fig. 5). The spatial distribution of these clusters has followed the spatial distribution of medical services for CS in the study area (Fig. 5). We also identified the space–time and spatial variations in temporal trends clusters of CS EBS rates in the study area based on space–time scan statistics. Our analysis showed significant spatial rate variations among the clusters, as reported in previous studies [36, 49]. In this study, cluster one experienced an average annual growth of 61.3%, but this growth was 3.56% in cluster two (Fig. 7). The high CS growth in cluster one can be due to the allocation of new maternal facilities to the hospital located in Dargaz county (Hospital No. 5) (Fig. 8B) in the region near cluster one. As a result, improving the spatial accessibility to CS facilities might have a considerable role in attracting optional CS demands in this area. Previous studies have also shown that CS rates in nearby hospitals have increased as the distance between hospitals and the place of residence of pregnant women has decreased [34, 35, 49]. For example, Harrison and Goldenberg showed that long distances to health facilities and poor transport systems had been documented as barriers to obtaining CS [50].

CS rates have fluctuated in the geographical areas of the study region at the study time and have decreased in comparison to the start point of the study time in 2016. This decrease can have several reasons; some studies have highlighted the effect of fear of COVID-19 infection on reducing CS on maternal request [51]. Furthermore, some researchers have noted that the implementation of governmental CS restriction guidelines since 2015 in Iran has reduced the rate of CS in public hospitals in recent years [52].

The interaction analysis of hospital facilities has shown that hospital care services have been significantly concentrated in particular areas of the study region and have followed a wholly clustered pattern (spatial dependence). This means an unequal distribution of hospital services care for CS deliveries in the study region (Fig. 8a). Similar studies have shown the same problem in most developing countries [4]. The highest number of referrals for CS was to hospitals concentrated in particular areas. i.e., southeast of KRP in the center of the province with the most equipped and high-quality hospitals. Previous studies have also confirmed that the rate of CS increases in well-equipped and high-capacity hospitals [36, 37, 53].

In future research, information on individual factors such as maternal health characteristics and socio-economic factors (e.g., family income level, mother education level, etc.) might be taken into account in mixed models of both spatial and individual-level analyses to reveal associated factors in regions with high CS rates. Although these hotspot areas might manifest a public health problem, low CS rates in the other sites would not mean that they have a more acceptable situation. Instead, it might reveal that these areas would not have appropriate access to adequate medical care. The women in these areas would have been forced to give natural birth, which could pose risks to the health of mothers and children in situations where CS has medical indications. Therefore, interventions can include improving spatial accessibility to CS services in areas with low accessibility to decrease maternal and child morbidity risk and reduce the inequity of CS-related health services.

The findings of this study showed that although the rate of CS is lower (35.39%) than the national average (47.9%) [54], it is higher than the global average (21%) [8] and the recommended rate of the WHO (10%-15%) [3, 9, 55]. Previous studies have confirmed similar results regarding the high rate of CS in Iran [38, 52]. The CS rate in this study is only for CS performed in public hospitals, while considering the number of CS in private hospitals might cause a higher rate [38]. The results of our study showed that the average age of mothers who used CS was 32.33 years, which confirms the results of previous studies showing that CS rates increase with age [17, 19, 20, 32]. Although the CS generally decreased during the last years in the study region, the rates are still higher than the world average. Our results can assist policymakers in implementing more targeted interventions for CS control and resource allocation in the most in-need areas. The present study reinforces the need for integrated prevention and control strategies of the unnecessary CS in the study region and regional policymakers might take long-term measures to decrease the CS through public awareness.

The present study vividly depicted the temporal, spatial, and spatiotemporal patterns of CS, with heterogeneous spatial patterns in the study region. We showed that integrating scan statistics with GIS visualization techniques is feasible to identify spatiotemporal patterns of CS. The approach can be generalized to other geographical areas as we used a simple dataset mentioned in additional data file.

### Policy implications

Policymakers might try to develop and implement some training programs in high clustered areas to reduce voluntary CS without medical indications. Considering that a high number of CS in the study area is optional [56], therefore, there is a need to inform mothers about the risks and consequences of CS through pre-pregnancy education. Based on our findings of the spatial interaction analysis, it was found that there is a kind of inequality in the spatial distribution of hospital facilities for CS in the study area. Consequently, health policymakers need to make suitable and accessible health facilities for mothers who need CS with medical indications. Improving spatial accessibility to the alternative hospitals and empowering the existing local hospitals to perform CS in areas out of service might be considered to decline spatial inequity in health care services.

### Limitations

We collected and analysed CS data from public hospitals and did not have private hospitals' data; this might be the underestimation of the CS rate distribution at the population level. However, this limitation would not cause a considerable difference in spatial results because both CS performed in private hospitals and government hospitals are covered by insurance in Iran. In other words, different economic statuses in different geographical areas would not cause any difference in the CS distribution among public and private hospitals. Furthermore, the CS data included both the elective and emergency CS, even though there were some regions with high CS density but that could also be due to the fact that those regions might have substantial progress in maternal and child health by saving women and children at risk during childbirth by CS. Another limitation can be using Euclidean distance as a proxy for travel times. However, as we did this study at the county level, not at the city level with heavy traffic, Euclidean distance would be strongly associated with travel times [57]. Using the number of all hospital staff, not the number of staff related to maternal care, would be another limitation in considering hospitals' capacity. In the end, we analyzed the data at the county level because the residential information was available at this level. A finer geographical level such as town could generate more reliable results.

## Conclusions

Both CS overutilization and lack of CS when it has medical indications are significant public health problems that healthcare policymakers might take into account. The CS clustered areas might be investigated to determine why they have lower or higher CS rates than the rest of the study area. Finally, equitable distribution of hospitals and reallocation of healthcare facilities is inevitable in the long-term in the study region to reinforce the regions for performing unintended and urgent CS.

## Supplementary Information

**Additional file 1: Table 1. ** Purely spatial detected clusters based on Poisson scanstatistic model for areas with high rates of C-section cases in RKP, Iran. **Table 2.** Retrospective Space-Time detected clusters based on Discrete Poisson model for areas with high rates of C-section cases in RKP, Iran. **Table 3.** Clusters of spatial variations in temporal trends for areas with high rates of C-section cases in RKP, Iran. 

**Additional file 2.**

**Additonal file 3.**

## Data Availability

All the data used in this study is publicly available via Additional file 3.

## Abbreviations

CS: Caesarean section
WHO: World Health Organization
EBS: Empirical Bayesian Smoothed
GMI: Global Moran's Index
GIS: Geographical information system
KDE: Kernel Density Estimation
OE: Observed/Expected
RR: Relative risk
LLR: Log Likelihood ratio
GI: Gravity intensity
