# Supplementary material for: Geospatial analysis of cesarean section in Iran (2016–2020): exploring clustered patterns and measuring spatial interactions of available health services
Source: BMC Pregnancy Childbirth. 2022 Jul 21;22:582. doi: 10.1186/s12884-022-04856-z (PMC9302231; doi:10.1186/s12884-022-04856-z)
Supplement: Supplementary file 1 — Additional file 1: Table 1. Purely spatial detected clusters based on Poisson scanstatistic model for areas with high rates of C-section cases in RKP, Iran. Table 2. Retrospective Space-Time detected clusters based on Discrete Poisson model for areas with high rates of C-section cases in RKP, Iran. Table 3. Clusters of spatial variations in temporal trends for areas with high rates of C-section cases in RKP, Iran. [file 12884_2022_4856_MOESM1_ESM.docx]

**Additional file**

Table 1: Purely spatial detected clusters based on Poisson ‎scan statistic model ‎for areas with high rates of C-section cases in RKP, Iran.‎

| Cluster name | Locations | Overlap with clusters | Coordinates / radius | Population | N. of cases | Expected cases | Annual cases / 100000 | Observed / expected | Relative risk | Log likelihood ratio | P-value |
| --- | --- | --- | --- | --- | --- | --- | --- | --- | --- | --- | --- |
| 1 | Taybad  Khaf | No Overlap | (34.411700 N, 60.032900 E) / 72.38 km | 68243 | 8567 | 2768.17 | 917026.7 | 3.09 | 3.37 | 4121.2 | < 0.001 |
| 2 | Bajestan  Bardaskan  Khalilabad  Kashmar  Kohsorkh  Mahvelat | No Overlap | (35.117900 N, 58.128000 E) / 71.69 km | 106966 | 9163 | 4338.91 | 625753.4 | 2.11 | 2.27 | 2196.01 | < 0.001 |
| 3 | Sarakhs | No Overlap | (36.291400 N, 60.781000 E) / 0 km | 26685 | 3564 | 1082.44 | 975622.5 | 3.29 | 3.41 | 1808.08 | < 0.001 |
| 4 | Chenaran  Dargaz  Quchan | No Overlap | (37.354900 N, 59.021600 E) / 68.28 km | 107731 | 6989 | 4369.94 | 473898.8 | 1.60 | 1.66 | 712.56 | < 0.001 |
| 5 | Zaveh  Fariman | No Overlap | (35.295300 N, 59.719500 E) / 46.67 km | 44186 | 2001 | 1792.34 | 330806.2 | 1.12 | 1.12 | 12.00 | < 0.001 |
| A cluster is statistically significant when its log likelihood ratio is greater than the  critical value, which is, for significance level:  Gumbel Critical Values:  0.00001: 13.902301  0.0001: 11.448543  Standard Monte Carlo Critical Values:  0.001: 10.484311  0.01: 6.221420  0.05: 4.999874 | | | | | | | | | | | |

Table 2: Retrospective Space-Time detected clusters based on Discrete Poisson model ‎for areas with high rates of C-section cases in RKP, Iran.‎

| Cluster name | Locations | Time frame | Coordinates / radius | Population | N. of cases | Expected cases | Annual cases / 100000 | Observed / expected | Relative risk | Log likelihood ratio | P-value |
| --- | --- | --- | --- | --- | --- | --- | --- | --- | --- | --- | --- |
| 1 | Taybad  Khaf | 2017 to 2018 | (34.411700 N, 60.032900 E) / 72.38 km | 68243 | 3578 | 1107.27 | 957488.4 | 3.23 | 3.34 | 1768.11 | < 0.001 |
| 2 | Bajestan  Bardaskan  Khalilabad  Kashmar  Kohsorkh  Mahvelat | 2016 to 2017 | (35.117900 N, 58.128000 E) / 71.69 km | 106966 | 4223 | 1735.57 | 720985.7 | 2.43 | 2.52 | 1310.84 | < 0.001 |
| 3 | Dargaz  Quchan | 2019 to 2020 | (37.354900 N, 59.021600 E) / 46.54 km | 66106 | 2201 | 1072.60 | 608037.7 | 2.05 | 2.08 | 462.48 | < 0.001 |
| 4 | Binalood  Chenaran  Golbahar | 2016 to 2017 | (36.547200 N, 59.196200 E) / 31.00 km | 83855 | 2042 | 1360.58 | 444711.2 | 1.50 | 1.51 | 150.86 | < 0.001 |
| A cluster is statistically significant when its log likelihood ratio is greater than the  critical value, which is, for significance level:  Gumbel Critical Values:  0.00001: 15.333440  0.0001: 12.862330  Standard Monte Carlo Critical Values:  0.001: 10.179902  0.01: 7.669345  0.05: 6.128707 | | | | | | | | | | | |

Table 3: Clusters of spatial variations in temporal trends for areas with high rates of ‎C-section cases in RKP, Iran.‎

| Cluster name | Locations | Coordinates / radius | Population | N. of cases | Expected cases | Annual cases / 100000 | Observed / expected | Relative risk | Inside time trend | Outside time trend | Log likelihood ratio | P-value |
| --- | --- | --- | --- | --- | --- | --- | --- | --- | --- | --- | --- | --- |
| 1 | Quchan | 37.117700 N, 58.588000 E) / 0 km | 46981 | 2110 | 1905.71 | 328073.8 | 1.11 | 1.11 | 61.322% annual increase | 2.375% annual decrease | 453.06 | < 0.001 |
| 2 | Bakharz  Taybad  Torbat-e-Jam  Khaf  Roshtkhar  Zaveh  Salehabad  Fariman | (35.037800 N, 60.296700 E) / 94.74 km | 227071 | 12536 | 9210.79 | 403281.8 | 1.36 | 1.43 | 3.558% annual increase | 2.007% annual decrease | 31.76 | 0.001 |
| A cluster is statistically significant when its log likelihood ratio is greater than the  critical value, which is, for significance level:  Gumbel Critical Values:  0.00001: 14.175942  0.0001: 11.772106  Standard Monte Carlo Critical Values:  0.001: 9.564302  0.01: 7.270689  0.05: 5.340004 | | | | | | | | | | | | |
